# Effect of Low-Load, Long-Duration Stretching on Pain and Mobility in Sacroiliac Joint Dysfunction

**DOI:** 10.7759/cureus.108987

**Published:** 2026-05-16

**Authors:** Sanya J Patel, Sandeep Shinde, Mebin S Thomas, Manoj P Ambali, Harshal Y Kale

**Affiliations:** 1 Musculoskeletal Sciences, Krishna College of Physiotherapy, Krishna Vishwa Vidyapeeth (Deemed to be University), Karad, IND; 2 Anatomy, Krishna Institute of Medial Sciences, Krishna Vishwa Vidyapeeth (Deemed to be University), Karad, IND; 3 Critical Care Medicine, Krishna Institute of Medial Sciences, Krishna Vishwa Vidyapeeth (Deemed to be University), Karad, IND

**Keywords:** leg length inequality, muscle stretching exercises, pain measurement, sacroiliac joint dysfunction, treatment outcome

## Abstract

Background: Sacroiliac joint dysfunction (SIJD) is a significant contributor to low back pain, often causing postural deviation and restricted mobility, disrupting daily life. In this study, a low-load, long-duration (LLLD) stretching protocol was compared to a conventional stretching protocol to evaluate the effects of each intervention. LLLD stretch is a burgeoning technique that applies sustained mild tensile force to augment tissue elasticity, dissipate pain, and enhance joint mobility.

Methods: This investigation employed a comparative study design enrolling 120 participants. The Numerical Pain Rating Scale (NPRS), provocative tests, Timed Up and Go (TUG) test, and limb length discrepancy (LLD) were employed as assessment tools in this study. The participants underwent a detailed protocol targeting the hamstrings, iliopsoas, piriformis, and thoracolumbar fascia. Statistical evaluation involved intragroup comparisons conducted using paired-samples t-tests. Intergroup differences in post-intervention outcomes were evaluated using a baseline-adjusted analysis of covariance (ANCOVA) to control for initial scores as a covariate.

Results: Following baseline adjustment, the experimental group demonstrated a greater reduction in pain (NPRS) compared to the control group (F = 65.11, p < 0.001, partial eta squared (ηp^2^) = 0.360), with an adjusted mean difference of 1.68. The correction of apparent LLD was also more effective in the experimental group (F = 136.43, p < 0.001, ηp^2 ^= 0.538). While both groups showed significant within-group improvements in functional mobility (TUG), no significant between-group difference was observed after adjusting for baseline values (p = 0.442).

Conclusion: The integrated LLLD stretching protocol is more effective for achieving pain relief and functional symmetry in SIJD compared to conventional stretching. However, improvements in general functional mobility remain comparable between the two intervention strategies.

## Introduction

The transmission of forces between the axial skeleton and the lower extremities is largely dependent on the sacroiliac joint (SIJ). The SIJ is a robust, weight-bearing synovial-amphiarthrodial articulation with little osteokinematic movement that is located between the sacrum and the ilium. The intrinsic stiffness required for load transfer during standing, walking, and dynamic functional activities is influenced by its bony structure and ligamentous stability [1]. The uneven facets on the joint surface improve congruency and help withstand shear stresses. The SIJ's contribution to lumbo-pelvic kinematics is crucial for maintaining effective posture, stability, and gait mechanics despite its limited passive motion, as noted by Vleeming et al. [2].

Strong ligamentous tissues, such as the interosseous sacroiliac ligament, sacrospinous ligament, and iliolumbar ligaments, anatomically support the SIJ. Together, these elements provide form closure, or the stability that comes from anatomical fit. Active muscle components, including the erector spinae and transverse abdominis, contribute to force closure alongside passive limitations, thereby improving functional stability through myofascial tension. While doing activities that require dynamic load sharing, such as ascending stairs, carrying weight, bending, and squatting, the joint performs well thanks to the special interplay of form and force closure [3].

Even though the SIJ is technically stable, changes in mobility, inflammatory alterations, postural dysfunction, trauma, muscle imbalance, or recurrent mechanical stress can make it a major cause of lumbopelvic discomfort. In general, discomfort or dysfunction originating from the SIJ and related periarticular tissues is referred to as sacroiliac joint dysfunction (SIJD). Localised sacroiliac pain, discomfort spreading to the groin or posterior leg, and functional restrictions during transitional movements are typical symptoms of SIJD. Clinically, SIJD can show up as either hypomobility or hypermobility. While hypomobility is more commonly linked to soft-tissue stiffness, asymmetric loading, or recurrent strain affecting the surrounding muscles and fascia, hypermobility can result from ligamentous laxity or hormonal factors associated with pregnancy [4].

SIJD impairs the effective transmission of loads between the pelvis and legs, from a pathomechanical standpoint. Compensatory mobility from nearby joints, such as the hips and lumbar, may be required due to altered or limited movement at the SIJ, which may ultimately result in secondary dysfunctions and chronic discomfort. Prolonged sitting, forward bending, ascending stairs, and unilateral loading tasks are frequently reported by patients as challenging. Mobility provocation tests are used in clinical evaluation because functional deficits are often accompanied by felt stiffness or localised soreness [4,5].

For SIJD, traditional physiotherapy methods usually focus on lumbopelvic stability, pain relief, and mobility restoration. Manual therapy methods, muscle energy techniques (METs), mobilisation, pelvic obliquity adjustment, therapeutic stretching, core and pelvic stabiliser strengthening exercises, neuromuscular re-education, and modalities such as heat and ultrasound for symptomatic relief are common [6-8]. These treatments can provide relief, but they don't always address the specific mobility limitations caused by the soft-tissue stiffness surrounding the SIJ. Tightness in the iliopsoas, rectus femoris, hamstrings, piriformis, or thoracolumbar fascia may cause altered pelvic posture in situations where hypomobility is predominant, perpetuating a cycle of discomfort and limited movement. Traditional stretching methods may be insufficient in providing prolonged mechanical stress necessary to influence deeper fascial tissues and are often limited by patient discomfort or protective muscle guarding [9,10].

An innovative treatment strategy called low-load, long-duration (LLLD) stretching aims to gradually alter connective tissue extensibility without causing pain or guarding reactions. LLLD delivers persistent low-intensity strain over extended durations, allowing for progressive tissue adaptation, different from dynamic or short-duration static stretching. The reasoning is justified by the viscoelastic behaviour of connective tissue, in which tissue elongation and creep are caused by ongoing mechanical stress [11,12]. Because of this, LLLD is especially appropriate for disorders requiring myofascial constriction, persistent tightness, or hypomobility in delicate anatomical areas. LLLD may be a useful addition to conventional physiotherapy regimens for those with SIJD, when mobility limitations lead to altered kinematics and discomfort [13].

However, research on utilising LLLD in the treatment of SIJD remains limited. Stability-centric exercises, core strengthening, and symptom-focused therapies are the main focus of conventional programs. While stability mechanisms are particularly relevant in patients presenting with hypermobility patterns, purely stability-based therapies may not be optimal for hypomobile SIJD cases. This discrepancy highlights a potential weakness in the present therapeutic approaches. Despite receiving regular physiotherapy care, many patients experience poor movement quality, stiffness, and functional restrictions. Additionally, therapy results are frequently inconsistent, in part because of differences in evaluation, intervention selection, and disregard for soft-tissue extensibility.

Recognising these limitations and the need to investigate alternative therapies to enhance tissue flexibility and joint mobility in SIJD led to the current investigation. The objective of this study was to compare the impact of LLLD stretching with conventional stretching in patients with SIJD. We hypothesised that the LLLD protocol would result in a greater reduction in pain and more pronounced improvements in functional symmetry compared to the control group. The primary outcome measure was pain intensity, while secondary outcomes included functional mobility and apparent limb length discrepancy (LLD).

## Materials and methods

This comparative study evaluated the effects of LLLD stretching on pain and mobility in SIJD. The research was carried out from January 22, 2026, to March 31, 2026.

Sample selection

The sample size for this investigation was determined a priori using a power analysis based on the primary outcome measure, the Numerical Pain Rating Scale (NPRS) [14]. Drawing from established parameters in recent clinical trial protocols for SIJD [15], the calculation was conducted to detect a clinically meaningful difference with an alpha level of 0.05 and a statistical power of 80%. The following formula was utilised: 

$N = \frac{(SD_1^2 + SD_2^2)(Z_{1-\alpha/2} + Z_{1-\beta})^2}{(\bar{x}_1 - \bar{x}_2)^2}$

This resulted in a minimum requirement of 55 participants per group (110 total). To account for an anticipated 10% attrition rate over the four-week intervention period, the final recruitment target was established at 120 participants (60 per group).

Initial screening evaluated 145 potential participants. Twenty-five individuals were subsequently excluded from the study, either for failing to meet the eligibility requirements (n = 18) or for refusing participation (n = 7).

To be eligible for the study, participants were required to be clinically diagnosed with SIJD, have a minimum pain duration of > 4 weeks, have an NPRS score of > 3/10, and be between the ages of 18 and 40 years. Furthermore, participants required a confirmed clinical diagnosis of SIJD.

The diagnostic threshold for SIJD was defined by the presence of localised sacroiliac pain and a positive response to a cluster of specific provocative and mobility tests:

Gaenslen’s Test

During the Gaenslen’s test (κ = 0.87; prevalence-adjusted bias-adjusted kappa (PABAK) = 0.88), the patient lies on his back near the edge. The test leg hangs off the plinth, while the contralateral leg is flexed towards the chest. One hand of the examiner stabilises the flexed knee while applying counterpressure at the extended leg. This stresses the SIJ, reproducing pain at the joint and indicating a positive test [16]. This test was utilised as a primary provocative measure to stress the SIJ.

Gillet's Test

For the Gillet’s test (κ = 0.314), the participant stands while the examiner palpates the posterior superior iliac spine (PSIS) with one thumb, and the other thumb palpates the S2 spinous process. Later, the participant flexes the leg of the same side being palpated. If the lateral thumb does not move downwards or moves upwards in reference to the thumb on S2, then the test suggests a positive sign [17]. This was utilised to assess aberrant SIJ mobility and functional dysfunction.

Alongside these, the distraction, compression, and thigh thrust tests were performed to ensure a minimum threshold of three positive provocative signs [18].

A strict differential diagnosis protocol was implemented to rule out other primary nociceptive sources:

Exclusion of Lumbar Pathology

Participants were evaluated using repeated lumbar flexion and extension movements; those demonstrating centralisation or peripheralisation of symptoms were excluded.

Exclusion of Hip Pathology (Flexion, Abduction, and External Rotation (FABER) Test)

While performing the FABER test (κ = 0.78; PABAK = 0.92), the patient lies supine with the test leg positioned in external rotation and knee flexion, resting the foot on the contralateral knee. The clinician stabilises the opposite anterior superior iliac spine (ASIS) with one hand while simultaneously exerting downward pressure on the flexed knee. The test is considered positive if the patient reports pain localised to the sacroiliac region on the side being evaluated [19]. If this test elicited pain specifically localised to the anterior hip or groin, or revealed restricted internal/external rotation, the participant was excluded to rule out primary hip joint pathology.

Finally, participants were excluded if they presented with neurological or psychological conditions, pregnancy, a history of pelvic fracture/surgery, or recent corticosteroid injections in the SIJ region. Consequently, 120 eligible participants were successfully enrolled.

Following baseline evaluations, the 120 enrolled participants were randomly assigned to either Group A (n = 60) or Group B (n = 60) using a simple randomisation method. To ensure rigorous allocation concealment, group assignments were contained within sequentially numbered, opaque, sealed envelopes (SNOSE). These envelopes were prepared by an independent researcher not involved in participant recruitment or clinical assessment. Each envelope was opened only after a participant’s eligibility was formally confirmed and baseline assessments were completed, ensuring treatment allocation remained completely concealed from the investigator until the point of formal enrollment.

Due to the inherent physical nature of the interventions, the present study utilised a single-blinded methodology. While the outcome assessors and treating clinicians were aware of the group assignments, the participants were strictly blinded. To mitigate performance bias, participants were kept entirely unaware of their specific group allocation, the overarching study hypotheses, and the protocols of the alternative group. Furthermore, strict clinical scheduling was enforced to prevent inter-group contact and avoid treatment contamination.

Ethical considerations

Before the initiation, approval for ethical clearance was taken from the Institutional Ethical Committee (IEC) of Krishna Vishwa Vidyapeeth (Deemed to be University), Karad, India (approval number: KVV/IEC/02/2026). Conducted in adherence to the ethical standards established by the Declaration of Helsinki, this study prioritised the protection of all human subjects. Each individual provided voluntary informed consent before joining the research, with a guarantee that their personal information would remain strictly confidential throughout the process. Furthermore, participants were explicitly advised that they could opt out of the study at any time without facing any negative consequences.

Outcome measures

NPRS

NPRS assessment (intraclass correlation coefficient (ICC) = 0.75-0.94) assesses the participant's pain intensity on a 0-10 linear scale, where ‘0’ indicates no pain and ‘10’ indicates intolerable pain. NPRS is a subjective measure of the intensity of the participant's pain [13].

Timed Up and Go (TUG) Test

The TUG test (ICC = 0.98) was used to quantify functional mobility. Participants were instructed to stand from a chair, walk the marked 3 m, turn, return to the chair, and sit back down on a verbal cue. Timing was recorded using a stopwatch in seconds. The shorter the time, the better the participant's functional mobility [20].

LLD

Tape-measured LLD (ICC = 0.78-0.99) was utilised as a secondary objective outcome to assess functional asymmetry associated with SIJD. Apparent LLD was taken from the umbilicus to the medial malleolus. A measurable discrepancy represents asymmetrical limb length influenced by pelvic tilt, sacral rotation, and SIJ stress [21]. To ensure standardisation and reproducibility, all final LLD values were strictly recorded and reported in centimetres.

Treatment protocol

Participants in both groups completed supervised physiotherapy sessions five days per week for a total of four consecutive weeks. To ensure reproducibility and patient safety, all sessions were conducted one-on-one under the direct supervision of a certified physiotherapist. Adherence was strictly monitored via daily clinic attendance logs, requiring a minimum of 90% compliance for inclusion in the final analysis.

In both groups, exercises were performed in a standardised sequence: (i) supine knee-to-chest, (ii) supine knee-to-opposite-chest, (iii) side-to-side knee, (iv) prone hamstring stretch, and (v) supine iliopsoas stretch. The stretching intensity for all participants was limited to a "strong but comfortable pull" (sub-painful threshold: < 4/10 on the NPRS) to prevent tissue guarding and symptom exacerbation.

Group A (LLLD Stretching)

**Figure 1:**
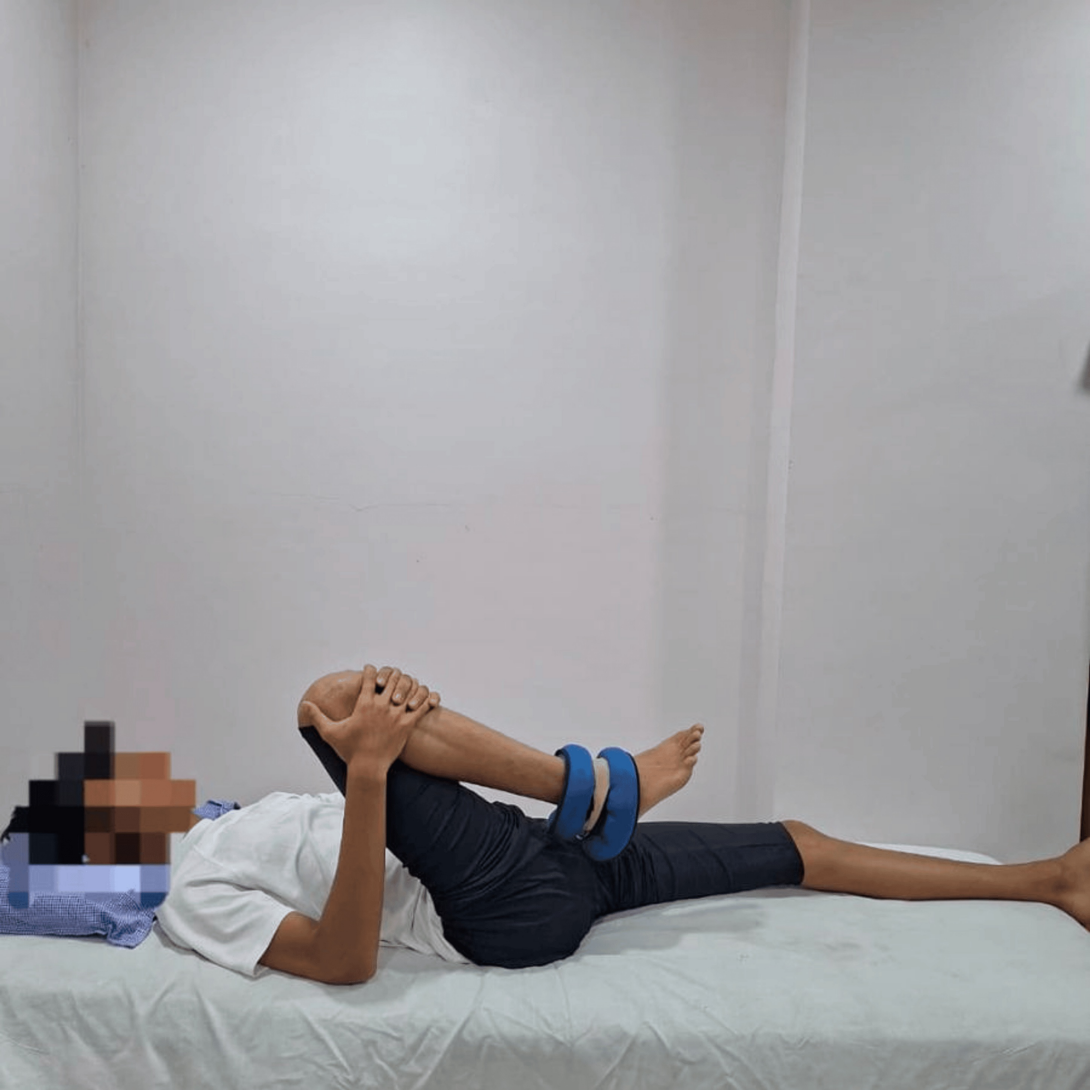
Single knee-to-chest LLLD stretching LLLD: low-load, long-duration

**Figure 2:**
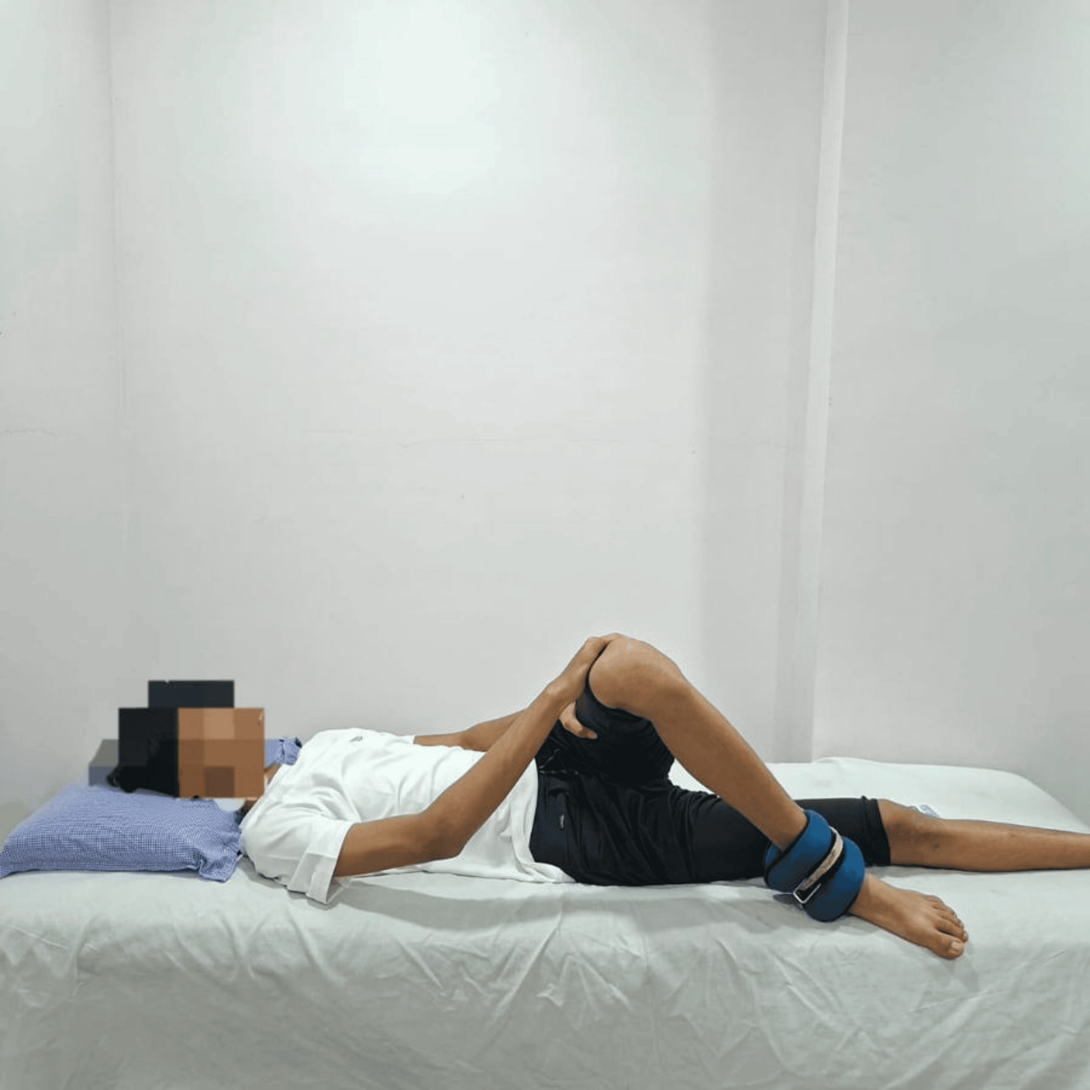
Knee-to-opposite shoulder LLLD stretching LLLD: low-load, long-duration

Participants in Group A performed the five standardised stretches utilising an external load to facilitate prolonged tissue creep. External loads (standardised physical therapy sandbags) were securely fastened using velcro straps just proximal to the joint line (distal femur) or at the distal tibia, depending on the specific stretch mechanics (Figures 1-2). Participants performed one continuous set per stretch per side. Because LLLD relies on uninterrupted mechanical tension, exercises were performed as one set of one continuous repetition per stretch. A standard 60-second rest interval was provided between different stretch types. The protocol progressed weekly as follows: (i) Week 1: One set of one repetition (60-second hold); 0.5 kg load. (ii) Week 2: One set of one repetition (90-second hold); 1.0 kg load. (iii) Week 3: One set of one repetition (120-second hold); 1.5 kg load. (iv) Week 4: One set of one repetition (150-second hold); 2.0 kg load.

Group B (Conventional Stretching)

Participants in Group B performed the identical sequence of five stretches without the application of external loads. A 15-second rest interval was provided between individual repetitions, and a 60-second rest interval was provided between different stretch types. The protocol progressed weekly by increasing repetitions and hold times: (i) Week 1: 15-second hold, performed for two repetitions per side. (ii) Week 2: 30-second hold, performed for three repetitions per side. (iii) Week 3: 45-second hold, performed for four repetitions per side. (iv) Week 4: 60-second hold, performed for five repetitions per side.

Statistical analyses

Statistical analyses were performed using IBM SPSS Statistics (IBM Corp., Armonk, USA). Descriptive statistics were calculated for baseline demographic and clinical characteristics. Baseline between-group comparisons were conducted using independent-samples t-tests, and effect sizes for baseline differences were reported using Cohen’s d. Within-group changes from pre-test to post-test were assessed using paired-samples t-tests. Between-group differences in the primary outcome (NPRS) and secondary outcomes (TUG test and apparent LLD) were evaluated using one-way analysis of covariance (ANCOVA), with post-test scores entered as dependent variables, treatment group as the fixed factor, and corresponding baseline scores as covariates. This approach was used to adjust for baseline imbalance and provide a more accurate estimate of treatment effects. Adjusted mean differences with 95% confidence intervals (CI), F-statistics, p-values, and effect sizes (partial eta squared (ηp^2^)) were reported for ANCOVA analyses. Statistical significance was set at p < 0.05.

## Results

A total of 120 participants fulfilled the study requirements and were included in the final data evaluation, with 60 participants in each group. No dropouts were reported during the four-week intervention period, and the study maintained a 100% completion rate with high adherence. Adherence was strictly monitored via daily clinic attendance logs. Importantly, no adverse events, such as significant pain exacerbation, muscle strain, or inflammatory responses, were reported during either the LLLD stretching or conventional stretching interventions, confirming the safety profile of both protocols.

Table 1 shows baseline demographic and clinical characteristics, including age, body mass index (BMI), symptom duration, affected side, and occupational factors, which were recorded for all participants. Most participants were aged between 26 and 32 years, with a slightly higher proportion of female individuals in both cohorts. A majority were classified as overweight (BMI ≥ 25.0 kg/m^2^) and reported a sedentary or desk-based occupational lifestyle. Statistical analysis utilising the Chi-square test revealed no significant between-group differences for age (p = 0.91), gender (p = 0.71), BMI (p = 0.70), side of symptoms (p = 0.92), or occupational activity levels (p = 0.70). Furthermore, symptom chronicity was highly comparable, with 53.3% of Group A and 50.0% of Group B reporting symptoms lasting between four and 12 weeks (p = 0.71), ensuring both groups started from a statistically similar baseline.

**Table 1:** Demographic variables of participants Data is presented as n (%). Statistical significance between groups was determined using a Chi-square test. A p-value > 0.05 indicates no statistically significant difference between the groups at baseline. Group A: LLLD stretching (n = 60); Group B: Conventional stretching (n = 60). LLLD: low-load, long-duration; BMI: body mass index

Variable	Group A	Group B
Age (years)		
18-25	20 (33.3%)	22 (36.7%)
26-32	25 (41.7%)	23 (38.3%)
33-40	15 (25.0%)	15 (25.0%)
Gender		
Male	30 (50%)	26 (43.3%)
Female	30 (50%)	34 (56.7%)
BMI		
Normal weight (< 25.0 kg/m^2^)	20 (33.3%)	22 (36.7%)
Overweight (≥ 25.0 kg/m^2^)	40 (66.7%)	38 (63.3%)
Duration of symptoms (pain chronicity)		
4-12 weeks	32 (53.3%)	30 (50.0%)
> 12 weeks	28 (46.7%)	30 (50.0%)
Side of symptoms		
Unilateral (right)	26 (43.3%)	25 (41.7%)
Unilateral (left)	22 (36.7%)	24 (40.0%)
Bilateral	12 (20.0%)	11 (18.3%)
Occupational factors/activity level		
Sedentary/desk-based	38 (63.3%)	40 (66.7%)
Active/manual/standing	22 (36.7%)	20 (33.3%)

In Table 2, a statistically significant baseline difference was observed in NPRS scores between the groups (p = 0.002), with Group A demonstrating higher initial pain levels. Therefore, baseline-adjusted ANCOVA was performed to control for this imbalance when evaluating post-intervention treatment effects. The baseline-adjusted analysis revealed significantly more pronounced improvements in the experimental group for pain and functional symmetry. For NPRS, Group A showed a significantly greater reduction in pain compared to Group B (F = 65.11, p < 0.001), with a large effect size (ηp^2^ = 0.360) and an adjusted mean difference of 1.68 (95% CI: 1.27 to 2.10).

**Table 2:** Baseline-adjusted comparison of outcomes between Group A and Group B using ANCOVA Values are expressed as mean ± SD. Between-group metrics were adjusted for baseline values using ANCOVA. Group A: LLLD stretching (n = 60); Group B: Conventional stretching (n = 60). ^† ^Indicates within-group significance calculated via paired-samples t-tests. ^‡ ^Indicates between-group significance and associated metrics (F-value, adjusted mean difference, and effect size reported as ηp²) calculated via one-way ANCOVA using baseline values as a covariate. Baseline between-group comparisons were performed using independent-samples t-tests, and effect sizes are reported as Cohen’s d. * Statistically significant at p < 0.05. SD: standard deviation; CI: confidence interval; NPRS: Numerical Pain Rating Scale; TUG: Timed Up and Go; LLD: limb length discrepancy; LLLD: low-load, long-duration; ANCOVA: analysis of covariance; ηp^2^: partial eta squared

Outcome measure	Time point	Group A (mean ± SD)	Group B (mean ± SD)	Within-group p-value^†^	Between-group adjusted mean difference (95% CI)^‡^	Between-group F-value^‡^	Between-group p-value^‡^	Effect size
NPRS (0-10)	Pre-test	6.23 ± 1.65	5.33 ± 1.37	_	0.90 (0.35 to 1.45)	10.57	0.002^*^	d = 0.60
	Post-test	2.83 ± 1.25	3.95 ± 1.60	< 0.001*	1.68 (1.27 to 2.10)	65.11	< 0.001*	ηp^2 ^= 0.360
TUG test (sec)	Pre-test	15.26 ± 3.33	14.62 ± 3.32	_	0.64 (−0.56 to 1.85)	1.11	0.294	d = 0.19
	Post-test	10.48 ± 2.54	9.90 ± 2.24	< 0.001*	-0.20 (-0.70 to 0.31)	0.60	0.442	ηp^2 ^= 0.006
Apparent LLD (cm)	Pre-test	2.05 ± 0.40	2.08 ± 0.38	_	−0.03 (−0.17 to 0.11)	0.18	0.674	d = 0.08
	Post-test	1.24 ± 0.43	1.73 ± 0.42	< 0.001*	0.47 (0.39 to 0.55)	136.43	< 0.001*	ηp^2 ^= 0.538

Similarly, the correction of apparent LLD was significantly greater in the experimental group (F = 136.43, p < 0.001, ηp^2^ = 0.538), with an adjusted mean difference of 0.47 cm (95% CI: 0.39 to 0.55). However, no significant between-group difference was observed for the TUG test after adjusting for baseline values (F = 0.60, p = 0.442, ηp^2^ = 0.006), as both groups demonstrated comparable functional improvements.

## Discussion

This research aimed to compare the impact of LLLD stretching with conventional stretching on pain and functional mobility in individuals with SIJD. A total of 120 participants were enrolled based on predefined inclusion criteria. Following a baseline-adjusted analysis (ANCOVA) conducted to control for initial differences between the groups, the results indicated that Group A demonstrated a more pronounced reduction in pain and greater improvement in functional pelvic alignment (apparent LLD) compared to Group B (p < 0.001). Regarding functional mobility as measured by the TUG test, while both groups showed significant improvements from baseline, the adjusted between-group analysis indicated comparable results between the two stretching protocols (p = 0.442).

Validity of pain reduction

A significant consideration in our findings was the baseline imbalance in pain scores, as the experimental group initially presented with higher NPRS values (6.23 ± 1.65) compared to the control group (5.33 ± 1.37). To address this, we employed a baseline-adjusted ANCOVA, which confirmed that the greater pain reduction in the experimental group was a robust treatment effect rather than a statistical artefact or regression to the mean. The analysis revealed a significant between-group difference (F = 65.11, p < 0.001) with an adjusted mean difference of 1.68 points (95% CI: 1.27 to 2.10). This confirms that integrated LLLD stretching provides a therapeutic benefit for pain relief that transcends the initial severity of symptoms.

Capsular stiffness, ligamentous tightness, and impaired neuromuscular control are frequently associated with SIJD discomfort, leading to aberrant load transfer across the pelvis. LLLD stretching enables viscoelastic deformation of connective tissues, allowing progressive extension without eliciting defensive muscle guarding or nociceptive reactions as reported by Zvetkova et al. [22]. Low-load persistent stretching has been shown in earlier research by Saito et al. to activate mechanoreceptors and decrease muscle spindle excitability, thereby lowering pain perception and increasing movement tolerance [23]. The extended period of stretch also favours plastic deformation rather than elastic rebound, resulting in enduring changes in tissue length and joint mechanics. This mechanism explains the reduction in the pain scores witnessed in the present study, aligning with findings reported by Matsuo et al., who highlighted the superiority of sustained stretching over short-duration stretches for pain modulation and tissue extensibility [24]. Further reducing aberrant shear stresses across the SIJ may be achieved by correcting pelvic asymmetry and improving sacroiliac alignment after LLLD stretching. The current results are corroborated by reports of similar pain-relieving benefits from prolonged stretching regimens in people with pelvic girdle dysfunction and persistent low back pain [25].

Functional mobility and the TUG test

Interestingly, while the experimental group achieved more effective results in pain and functional symmetry, both groups demonstrated similar improvements in functional speed, as measured by the TUG test (p = 0.442). This suggests that while LLLD may provide superior plastic deformation for tissue length, the functional task of rising from a chair and walking appears to benefit equally from either stretching methodology in this population. SIJD frequently results in altered gait patterns, delayed transitional movements, and decreased confidence when performing ambulation and sit-to-stand exercises. Pain, pelvic asymmetry, and poor load transfer are often the secondary causes of these functional impairments [26]. By improving muscle length-tension relationships, restoring pelvic mobility, and enabling more effective activation of stabilising muscles during functional activities, LLLD stretching probably enhances TUG performance. Smoother movement transitions and better postural control are made possible by LLLD stretching, which lessens stiffness in muscles such as the iliopsoas, hamstrings, and sacroiliac ligaments. Functional mobility can be positively affected by pain reduction alone, as demonstrated by Kwan et al. [27], who found a clear correlation between lower pain levels and better TUG performance. However, the improvement shown in this study may go beyond pain modulation and represent biomechanical rectification. Similar gains in functional mobility have been noted in studies assessing conservative treatments for SIJD and persistent low back pain that target restoration of pelvic symmetry and flexibility [28].

Clinical impact on symmetry

One important outcome of the present investigation was a high F-value of 136.43 and a large effect size (ηp^2 ^= 0.538), which indicated that the intervention was highly effective at addressing the perceived discrepancy. It is critical to clarify that these observed reductions did not reflect an anatomical structural alteration or lengthening of the osseous components, which would have been physiologically improbable within a four-week timeframe. Rather, the changes reflected a correction of functional pelvic asymmetry. SIJD frequently presents with anterior or posterior innominate rotations driven by myofascial imbalances (e.g., adaptive shortening of the iliopsoas or hamstrings). These rotations alter the spatial orientation of the ASIS. The targeted stretching protocols, particularly the prolonged tissue creep achieved in the LLLD group, effectively mitigate these muscular restrictions, restoring neutral pelvic alignment and consequently resolving the functional LLD. These differences in myofascial imbalances result in persistent discomfort and dysfunction, in addition to aberrant gait mechanics, as reported by Qureshi et al. [29]. The adaptive shortening of soft tissues that sustain pelvic malalignment is the focus of LLLD stretching. To promote symmetrical pelvic posture, controlled connective tissue remodelling was enabled by the progressive application of increasing loads (0.5 kg to 2 kg over four weeks). This is compatible with the principles of creep and stress relaxation stated in the connective tissue biomechanics literature [30]. The reported correction of functional limb-length disparities following extended stretching and manual treatment is supported by the observed decrease in LLD. The benefits in pain and mobility outcomes are reinforced by the restoration of pelvic symmetry, which also improves the length of limbs and load distribution throughout the SIJ [31].

Strengths, limitations, and future recommendations

This study aimed to highlight the impact of LLLD stretching compared to conventional stretching, without the inclusion of any additional physiotherapy interventions. The focus was on evaluating the benefits of LLLD stretching alone for pain intensity and functional mobility in individuals with SIJD.

For the management of SIJD, a direct comparison of two physiotherapy methods yielded clinically useful information. Internal consistency and interpretability were improved when validated and commonly used clinical outcome measures were employed. A more thorough assessment of therapy response was achieved by including both subjective (NPRS) and objective (TUG and apparent LLD) outcomes. Consistency among participants was guaranteed via a structured intervention approach. Additionally, both groups showed improvements in pain and functional mobility that were clinically significant, demonstrating the need for conservative physical treatment for SIJD. Furthermore, the application of a baseline-adjusted ANCOVA ensured that the findings were statistically rigorous and accounted for initial symptom severity, addressing potential biases in recovery magnitude. Finally, the focus on cost-effective, non-invasive techniques supports the translational applicability of these findings to routine physiotherapy practice.

The present study has several limitations that warrant consideration. Primarily, due to logistical constraints, the trial utilised a single-blinded design. A specific limitation of this study was the observed baseline imbalance in NPRS scores, as the experimental group initially reported higher pain levels than the control group. While this disparity was addressed through a baseline-adjusted ANCOVA to isolate the true treatment effect, the higher initial severity in the experimental group may still influence the interpretation of the magnitude of change. Patient-reported outcomes (NPRS) minimise direct influence; unblinded assessors may introduce measurement bias in objective secondary measures (TUG, LLD). Additionally, the four-week intervention period and absence of longitudinal follow-up preclude the assessment of long-term adaptations, durability of gains, or recurrence rates. Furthermore, outcome parameters were restricted to clinical pain and mobility scales. While apparent LLD is a practical clinical measure, manual assessments are subject to greater potential for examiner error than radiographic techniques, limiting the mechanistic understanding of biomechanical alterations. Fourth, the absence of a pure no-treatment control group restricts the ability to definitively isolate intervention effects from spontaneous healing or placebo responses. Finally, the observation of functional parity in the TUG test suggests that while this measure is effective for assessing general mobility, it may lack the sensitivity required to capture nuanced biomechanical changes specific to the integrated neuromuscular protocol. Future investigations should employ double-blinded designs, long-term follow-ups, instrumented objective measures, and precise subgroup stratifications.

Larger, more varied cohorts should be used in future research to increase statistical robustness and generalisability. To assess pain retention, mobility, and alignment improvements, long-term follow-up is advised. Stabilisation activities and core training should be investigated in conjunction with LLLD stretching since they may have synergistic benefits. Motion capture and force plates are examples of biomechanical or gait analysis technologies that may be used to clarify the neuromuscular mechanisms underpinning improvements. Causal inference may be strengthened by including a control group intervention.

## Conclusions

In conclusion, this four-week study indicates that the LLLD stretching protocol leads to more pronounced improvements in pain and functional symmetry for individuals with SIJD. By utilising a baseline-adjusted ANCOVA, we confirmed that the therapeutic impact on pain and apparent LLD was independent of the initial symptom severity. While both the LLLD and conventional stretching protocols were effective in enhancing general functional mobility, as measured by the TUG test, the LLLD approach was more effective in addressing the underlying mechanical drivers of the condition. These findings suggest that for patients where functional symmetry alignment and specific pain relief are the primary clinical goals, this specialised stretching approach provides an enhanced therapeutic outcome over conventional methods alone.
